# Developing and Costing Local Strategies to Improve Maternal and Child Health: The Investment Case Framework

**DOI:** 10.1371/journal.pmed.1001282

**Published:** 2012-08-07

**Authors:** Eliana Jimenez Soto, Sophie La Vincente, Andrew Clark, Sonja Firth, Alison Morgan, Zoe Dettrick, Prarthna Dayal, Bernardino M. Aldaba, Beena Varghese, Laksono Trisnantoro, Yogendra Prasai, Investment Case Team for India, Indonesia, Nepal, Papua New Guinea and the Philippines, Anna Bauze, Deni Harbianto, Soewarta Kosen, Aleli Kraft, Faozi Kurniawan, Michelle D. Macalintal, Paul A. Mariano, Kim Nguyen, Marian Angelika K. Panganiban, Rajashree Panicker, Johanna M. Santiago, Sneha Singh, Jacobe J. Villareal, Yulia Widiati

**Affiliations:** Burnet Institute, Australia; Gadjah Mada University, Indonesia; National Institute of Health and Development, Indonesia; UPecon Foundation, Philippines; Gadjah Mada University, Indonesia; UPecon Foundation, Philippines; UPecon Foundation, Philippines; School of Population Health, University of Queensland, Australia; UPecon Foundation, Philippines; Public Health Foundation of India; UPecon Foundation, Philippines; Public Health Foundation of India; UPecon Foundation, Philippines; Gadjah Mada University, Indonesia; 1 School of Population Health, University of Queensland, Herston, Queensland, Australia; 2 Centre for International Child Health, Murdoch Childrens Research Institute, University of Melbourne, Royal Children's Hospital, Parkville, Victoria, Australia; 3 Department of Health Services Research and Policy, London School of Hygiene & Tropical Medicine, London, United Kingdom; 4 Nossal Institute for Global Health, University of Melbourne, Carlton, Victoria, Australia; 5 UPecon Foundation, Inc., UP School of Economics, University of the Philippines, Diliman, Quezon City, Philippines; 6 Public Health Foundation of India (PHFI), Vasant Kunj, New Delhi, India; 7 Centre for Health Service Management, Faculty of Medicine, Gadjah Mada University, Jogjakarta, Indonesia; 8 New ERA, Rudramati Marg, Kalo Pul, Kathmandu, Nepal

## Abstract

Eliana Jimenez Soto and colleagues describe the Investment Case framework, a health systems research approach for planning and budgeting, and detail the implementation of the framework in four Asian countries to improve maternal, newborn and child health.

**Summary Points**

- At the sub-national level—where most health services are delivered—critical knowledge and capacity gaps exist, which prevent evidence from making a direct contribution to health plans and budgets.
- The Investment Case framework pairs locally led problem-solving analysis with quantitative techniques to inform local planning and decision-making.
- The framework allows for the development of locally appropriate strategies to overcome identified health system constraints and it estimates cost and impact should such strategies be implemented.
- The varied success of this initiative in terms of influencing annual plans and budgets reflects the political nature of resource allocation and the need to embed such approaches in the local policy process.
- To sustain evidence-based planning, we propose a collaborative arrangement that allows researchers to address specific evidence gaps and health managers to focus on their core business of delivering universal health coverage.

## Background

Technically feasible and cost-effective interventions exist to reduce maternal, newborn, and child mortality [1],[2]. This potential has not been fully realised due to the failure of health systems to improve the delivery and uptake of these priority interventions, particularly amongst the most vulnerable women and children. Underfunded investments in maternal, newborn, and child health (MNCH) are part of the impediment [3],[4], but unspent funds in a diversity of resource-constrained settings reflect a common problem of low absorptive capacity and the challenges of implementation at the local level [5],[6],[7],[8]. Health systems research to understand the impediments to scaling-up these cost-effective interventions is critical in resource-poor settings but is rarely prioritised [9], with much of the research that does exist focused at the global or national level [10],[11].

**Figure 1:**
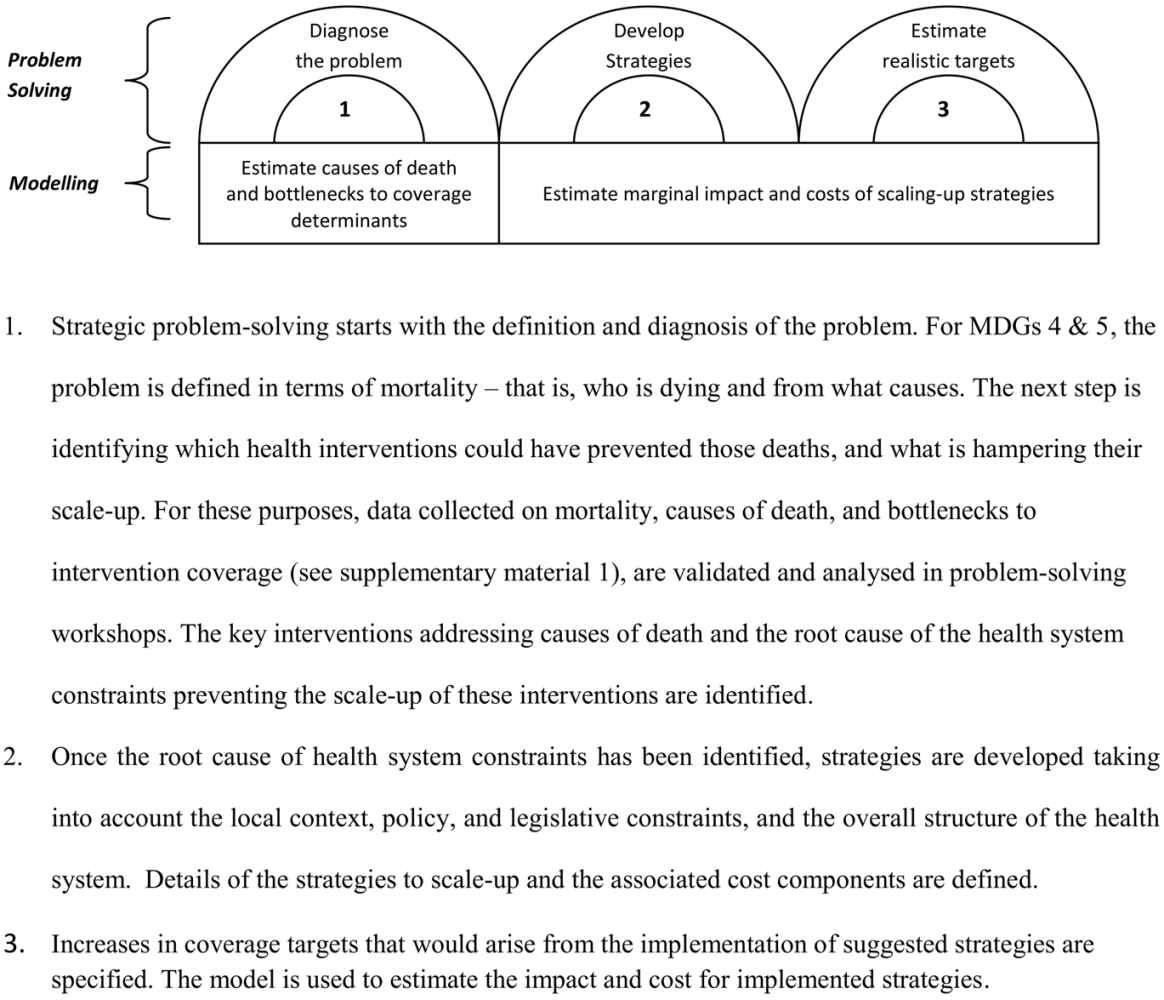
The Investment Case framework. MDG, Millennium Development Goal.

The Investment Case (IC) framework is one such health systems research approach that aims to support MNCH planning and budgeting. We do this by working with local planners and stakeholders to (i) identify the local constraints hampering the scaling-up of cost-effective MNCH interventions; (ii) design realistic strategies to address those constraints; and (iii) estimate the expected mortality impact and costs of implementing strategies. The framework (Figure 1) combines strategic problem-solving [12] with a decision-support model. Since the approach includes estimates of cost and impact of implementing strategies, the expectation is that it can not only be used by local planners to produce evidence-based plans linked to budgets, but also to advocate for more and better allocated funding towards MNCH.

We implemented this approach in four Asian countries: India, Indonesia, Nepal, and the Philippines. In our aim to influence plans and budgets, the success of the IC was varied and reflected the difficulties of managing decentralised systems. Here we detail the implementation of the framework in the four participating countries, how the process was used to influence planning and budgeting in each setting, and the lessons learnt.

## Choice of Study Site

To ensure the IC would be able to inform the development of MNCH plans and budgets, it was important to undertake analysis at the level where health planning takes place. In devolved settings, policymakers opted for IC development in a few locations representative of “typical” disadvantaged sub-national units. This included two districts and two cities in Indonesia, two provinces and one city in the Philippines, and two districts in the State of Orissa, India. In the centralised health system of Nepal, three clusters of disadvantaged districts were chosen that represent the country's different ecological regions.

## The Investment Case Framework

The IC framework pairs locally led problem-solving analysis with robust quantitative techniques to inform local decision-making (Figure 1). The range of data required for performance-based planning and budgeting is often not readily available, and must be reconciled and integrated for use in the development of local plans and budgets. The IC performs this function by bringing into a single framework the various streams of evidence answering the key research questions underlying evidence-based plans and budgets: (i) demographic/epidemiological—who is dying and from what causes, which interventions would be most effective at addressing the burden of mortality and what is their current coverage; (ii) implementation science/health systems—what are the health system constraints to scaling up those interventions and which are the most effective strategies to remove those constraints; and (iii) economic and financial—what resources are available and how much would it cost to implement the scaling-up strategies.

Strategic problem-solving starts with the definition and diagnosis of the problem. The IC team at each site started by mapping available local data on mortality, health intervention coverage, and health system costs. Once the available evidence was collected and evaluated, problem-solving workshops were undertaken. The composition and numbers of participants at these workshops differed in each country but included health officials from various levels of government, field workers, MNCH experts, and development partners. These facilitated workshops were each run over two to three days, and followed a standard format. This IC used the bottlenecks approach, which was originally developed by Tanahashi [13] and further refined by UNICEF and The World Bank [14], to aid a systematic diagnosis of health system constraints and formulation of strategies (Text S1). It provides a consistent and structured way to help local stakeholders evaluate the major determinants of health intervention coverage and identify the root causes of the health system constraints in disadvantaged locations. Strategies to address these constraints were developed taking into account the local context, policy, and legislative constraints, and the overall structure of the health system. Coverage targets that could be achieved if identified strategies were implemented were also estimated. Since the objective was to formulate strategies that can be implemented by the health sector in the next budgeting cycle, the emphasis was on improvements in health services within the current policy framework. A limitation of such a pragmatic focus is that high level policy options such as the introduction of social insurance are not taken into consideration.

Performance-based plans and budgets require an understanding of the expected impact and costs of alternative strategies proposed. We thus developed an Excel-based decision-support model that estimates the expected marginal impact and costs of implementing alternative scenarios of scale-up strategies. To estimate the expected impact that coverage targets would have on health outcomes, we use available evidence from the literature [1],[2],[10],[15]. Such evidence, which is limited to the efficacy of critical interventions on causes of death, led us to choose mortality indicators as a measure of impact. Acknowledging the difficulties tracking mortality at sub-national level, impact measures were used not to monitor progress but rather to indicate the likely benefits of one scenario over another. For example, impact estimates were used to illustrate that increasing coverage of emergency obstetric care would lead to substantial reductions in maternal mortality due to post-partum haemorrhage.

Results of the problem-solving analysis and the modelling of cost and impact for the four countries are presented elsewhere (E. Jimenez-Soto, S. La Vincente, A. Clark, S. Firth, A. Morgan, et al., unpublished observations). To illustrate the types of constraints and strategies arising from the problem solving analysis as well as the impact and cost of implementing these strategies, Text S2 provides a case study from Nepal. To assess the policy impact of this initiative in the year 2011, a comparison was made between the IC strategies and those adopted in the plans and budgets that the IC intended to influence.

## Did the IC Approach Influence Planning and Budgeting?

In Orissa, since the strategies identified in the two sites were relevant to other districts and required state-level action, the results of the IC were used to inform the development of the state level 2011–2012 National Rural Health Mission Programme Implementation Plan. Some of the key IC strategies were already included in the previous year's state plan. However, new strategies were identified that focused on simple solutions to address implementation bottlenecks. For example, to prevent “doubling up” of duties and improve availability of health personnel, the IC recommendation was to divide the responsibilities between cadres of the same field staff located in the same area. The IC also revealed that district officials were unaware of state-level strategies and policies recognized by all parties as important to scale-up coverage. This information prompted the state government to improve their communication channels with districts and put in place mechanisms to support implementation. For state officials, the use of the IC costing estimates also provided a powerful rationale for disadvantaged districts with smaller populations to advocate for budget allocation based on needs rather than population.

In the Philippines, the IC process influenced the development of annual plans and budgets in all three local government units, with incorporation of the recommended IC strategies. The IC findings, in particular the results of the problem-solving analysis and the costing information, were found to be very helpful in developing the various inputs for the Annual Operations Plans (AOPs) and negotiating budget allocations with other authorities. In one of the sites, prior to the IC, an urban health infrastructure strategy was under consideration by health system planners. The IC modelling showed that this strategy would have a minimal impact on MNCH, despite having a high cost. These IC findings provided convincing evidence in an accessible format, which could be used to lobby for an alternative focus on innovative public-private partnerships. These strategies were endorsed and funded through the local implementation plans for 2011. We are currently assessing the extent to which these strategies have been implemented and kept in the subsequent annual plans for 2012. The team has been in discussions with the government regarding the use of a simplified IC methodology in other areas of the country.

The IC in Nepal occurred in parallel to the development of the five-year National Health Sector Programme – Implementation Plan II (NHSP-IPII) and focused on clusters of disadvantaged districts. Political circumstances along with unrealistic timelines prevented the IC results from having a direct influence on planning and budgeting processes. However, national government officials involved in the IC noted the potential added value of the IC approach to support individual district-level evidence-based plans and budgets, particularly within the current discussions of decentralised planning. A district level IC is currently underway, to examine the extent to which this approach can be used to mobilise local resources, strengthen local capacity for problem-solving, and influence central government resource allocation.

In Indonesia, local plans in the four sites have included a large number of the IC recommendations that are within the domain of district health offices. However, we found that a large number of strategies required to scale-up critical MNCH services—such as those related to family planning or comprehensive emergency obstetric and neonatal care—were not adopted, as they involve high transaction costs and face coordination and funding constraints at higher levels of government. The evidence provided by the IC in this respect has prompted national policymakers participating in the IC Steering Committee to investigate feasible ways of addressing these high-level issues within the current political environment. In Papua—where there is greater autonomy over fiscal resources—the IC is currently being used with a different focus, that is, to assess whether it can contribute to more efficient allocation of resources in this province.

## Lessons Learnt

### 1. Using Evidence to Influence Policy Processes

The approach aims to address the technical demands of planning and budgeting, with the expectation that through use of evidence, planners will be better able to influence the political dialogue for resource allocation. Governments in our study sites acknowledged the utility of the process and supported further work, but our varying degree of success demonstrates that evidence-based initiatives like the IC need to be strongly anchored in policy processes directly aimed to strengthen local planning and budgeting. Such processes are political by nature and the IC framework does not directly capture those political elements. A retrospective evaluation of the IC, currently underway, will help to unpack those factors and elicit the extent to which other policy frameworks addressing the political aspects of priority-setting can aid the process.

### 2. Engagement of Policymakers and Fitting in with Government Timelines

**Figure 2:**
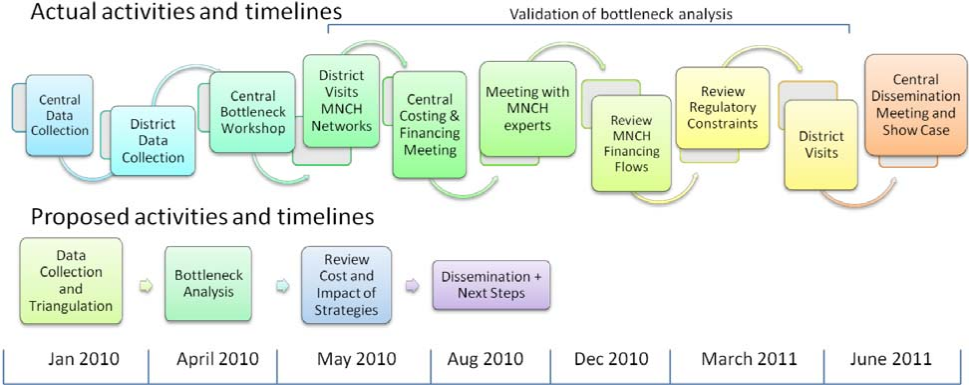
Comparison of proposed and actual timeline of IC activities in Indonesia.

In a planning support activity such as the IC—which seeks to work in partnership with governments and inform the planning process—relationships with key stakeholders are critical. The extensive engagement with policymakers necessary for these sorts of initiatives is both a strength and a limitation. Local planners want to understand the implications of their decisions, and local-level problem-solving allows for genuine “bottom up” strategic planning, empowering those delivering services to “own” intervention coverage targets. However, such levels of stakeholder engagement required time and resources. The need for IC results to feed into and complement existing planning and budgeting processes and timeframes and for the team to respond promptly to requests from government also required a flexible approach in each country. For example, Figure 2 illustrates the case of Indonesia, where at the request of government officials, multiple new activities were included to facilitate the policy dialogue with various sectors and levels of government.

### 3. Addressing Poor Quality Data at a Local Level

The current ICs have relied on intensive and costly one-off data collection and validation exercises. One of the key challenges encountered was the limited availability of high-quality data, even for basic parameters such as intervention coverage, at the sub-national level. In such cases available data at higher levels (e.g., state) were used after local validation of estimates to ensure that these reflected the local situation, and were accepted by local stakeholders. Continuing investments in health information systems are required to improve the quality of routine data and create direct links to evidence-based sub-national policy exercises like the IC. Our experience suggests that once they are shown the utility of local data, local planners are keen to strengthen their routine data collection systems, and request guidance in how to achieve this.

### 4. Maintaining the Quality of Problem-Solving Discussions

The IC process was well-received by health planners as a framework that allowed systematic examination of their data and formulation of achievable targets relevant to identified local health system constraints. There is limited evidence for which strategies are the best to scale up critical interventions and the evidence that does exist is highly context-specific, with the impact of different approaches strongly influenced by multiple factors such as implementation design, politics, and governance (S. Hollingworth, D. Hertz, A. Malik, S. Forsyth, E. Jimenez-Soto, unpublished observations). This lack of evidence makes the quality of discussion during the problem-solving workshops even more critical, and without due attention this can be a potential limitation of such processes. Particular attention was paid to facilitation of these workshops to preserve the quality of the discussions. Strategies identified during the problem-solving workshops were subsequently reviewed by MNCH experts, for further validation, to introduce novel or innovative approaches not previously considered by workshop participants and to advise whether proposed strategies would work within the current regulatory environment.

### 5. The Use of Impact and Costing Estimates

The estimation of impact and costs was found to be a particularly useful aspect of the IC process, with the modelling exercise used as an aid rather than a substitute for sound problem-solving. These results can help to guide decisions around selection and prioritisation of strategies, which can then be used to make the case to administrators and funders. However, the complexity of the modelling exercise posed challenges to capacity building. In some instances, opportunities for policy dialogue were missed because results could not be provided at very short notice or on the spot. As a result, we have developed a simplified version of the decision-support model, which is being used in the new IC sites.

## Conclusion

Recent studies suggest that, in resource-limited settings, priority should be given to health policy and systems research to improve coverage of existing effective MNCH interventions [9]. There is also a call to build and support the capacity of local experts and policymakers to engage in priority-setting exercises using the best available tools and evidence [16]. The IC approach aims to improve up-take of critical MNCH interventions by improving local capacity for planning and budgeting using a robust methodology. Whilst the success of this initiative has been varied across our study sites, the positive feedback received from key stakeholders suggests an untapped potential for the use of similar knowledge-sharing approaches to support policy dialogue in-country. We found the multi-partner approach, whereby the research team partnered with health planners, to be an effective model. This allows researchers to address specific research gaps and allows the health managers to focus on their core business of delivering universal health coverage.

## Supporting Information

Text S1 **The bottlenecks approach.** Explanation of the bottlenecks approach used for the Investment Case Project.
(DOC)

Text S2 **Nepal case study.**
(DOC)

## Abbreviations

IC: Investment Case
MNCH: maternal, newborn, and child health
