# Supplementary material for: Developing and Costing Local Strategies to Improve Maternal and Child Health: The Investment Case Framework
Source: PLoS Med. 2012 Aug 7;9(8):e1001282. doi: 10.1371/journal.pmed.1001282 (PMC3413720; doi:10.1371/journal.pmed.1001282)
Supplement: Text S1 — The bottlenecks approach. Explanation of the bottlenecks approach used for the Investment Case Project. (DOC) [file pmed.1001282.s001.doc]

**The Bottlenecks Approach**

The term bottleneck is used to define particular elements that limit a whole system’s capacity to improve the health outcomes of the population. In our methodology the health system is examined against a range of supply, demand, and quality factors that determine the extent to which the population benefit from health services. The analysis rationale is based on the work of Tanahashi (1), subsequently adapted by Soucat and colleagues in the early 2000s (2).

Tanahashi proposes five coverage determinants of health services: availability of staff, supplies, and facilities; geographical accessibility; acceptability by users as determined by cost and other demand side factors; contact coverage, that is, actual use of the services by the target population; and effectiveness coverage as guaranteed by the quality of services provided (1). The application of this approach presents some measurement challenges. It is difficult to combine staff, supplies, and facilities in a single indicator. Acceptability cannot be directly observed so its measurement would require a theoretical construct that would capture the range of factors that affect acceptability of services. Without being able to quantify individual coverage determinants, the analysis remains theoretical, and is of less use to local health planners.

Soucat *et al* have overcome these measurement challenges by further disaggregating availability into commodities and human resources, and proposing contact coverage as a measure of revealed acceptability. For complex interventions involving a package of services, such as antenatal care or immunisation, an additional indicator, continuous use, has also been included. Webfigure 1 provides an example of coverage determinants for Antenatal Care (ANC) in the Sikka district of Indonesia.

**Webfigure 1: Example of Coverage determinants at baseline for Antenatal care in the Sikka district of Indonesia**


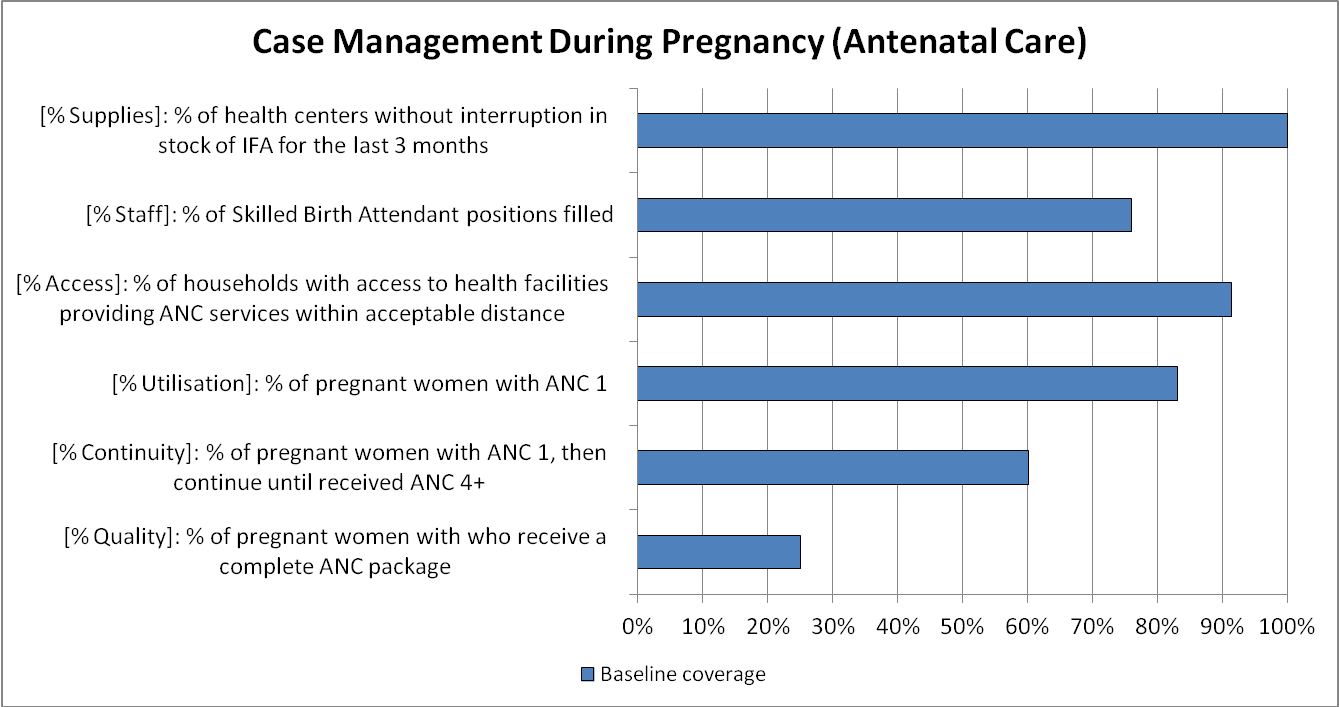


IFA refers to Iron-Folate tablets, ANC 1 refers to first ANC visit, ANC 4 refers to fourth ANC visit.

**References**

1. Tanahashi T. Health service coverage and its evaluation. Bulletin of the World Health Organization. 1978;56(2):295-303.

2. Soucat A, Lerberghe W, Doip F, Nguyern S, Knippenberg R. Marginal budgeting for bottlenecks: A new costing and resource allocation practice to buy health results. Draft Report. Washington DC: World Bank, Institute of Tropical Medicine, UNICEF, WHO 2002.
