# Supplementary material for: Developing and Costing Local Strategies to Improve Maternal and Child Health: The Investment Case Framework
Source: PLoS Med. 2012 Aug 7;9(8):e1001282. doi: 10.1371/journal.pmed.1001282 (PMC3413720; doi:10.1371/journal.pmed.1001282)
Supplement: Text S2 — Nepal case study. (DOC) [file pmed.1001282.s002.doc]

**Nepal case study**

Nepal – Adapting national plans to the local context

The IC examined the constraints to quality coverage of MNCH interventions in the most disadvantaged districts of the three eco-regions of Nepal. As shown in the table below, the IC specific strategies that addressed the local context have the potential to achieve relatively similar impact at less cost when compared with the comprehensive national strategies proposed by Nepal Health Sector Programme - Implementation Plan II (NHSP -IPII) For example, for the hill and mountain regions, the IC strategies focused on improved access through:

- additional supervision and revitalization of volunteer led mothers’ groups;
- support for increased outreach visits by providing secure accommodation to female outreach staff;
- increasing the range of family planning options and improving logistics management to ensure the supply of essential MNCH drugs;
- selected facility upgrades for Basic Emergency Obstetric Care service provision and 24/7 access to skilled birth attendants

In addition, the NHSP-IP II strategies demanded increases in human resources and infrastructure resulting in higher costs, namely:

- proposed maternity waiting homes in hill and mountain districts
- a threefold increase in the number of new Primary Health Care Centres in the terai, to meet new population based health service ratios.

|  | IC | NHSP- IP II |
| --- | --- | --- |
| Per Capita Marginal Cost* | $2.11 (Terai/Plains)  $2.17 (Hill)  $4.08 (Mountain) | $4.56 (Terai/Plains)  $3.15 (Hill)  $5.00 (Mountain) |
| Estimated reduction in MMR | 23% (Terai/Plains)  34% (Hill)  26% (Mountain) | 31% (Terai/Plains)  40% (Hill)  40% (Mountain) |
| Estimated reduction in U5MR | 18% (Terai/Plains)  33% (Hill)  24% (Mountain) | 20% (Terai/Plains)  36% (Hill)  31% (Mountain) |

*Annualised over 5 year period

The other notable difference between the NHSP-IPII and the sub-national analysis was in the estimates of achievable coverage targets. Since disadvantaged districts start from a lower baseline, these national targets may not be realistic. For example the baseline coverage of met need for emergency obstetric care is between 1-6% in the IC districts versus 31% at national level. The targets chosen at the IC problem-solving workshop of 20% might therefore be more feasible for these disadvantaged districts than the current national target of 49%.

The NHSP-IPII is a strong evidenced based plan, as yet not fully funded, and the IC can assist in informing difficult decisions about which national strategies are best suited to particular contexts.
